# Asymmetry of the male internal reproductive organs in Mantophasmatodea

**DOI:** 10.1186/s40850-021-00105-6

**Published:** 2022-01-07

**Authors:** Josefine Kreuz, Monika J. B. Eberhard

**Affiliations:** Zoological Institute and Museum, General Zoology and Zoological Systematics, University of Greifswald, Loitzer Str. 26, 17489 Greifswald, Germany

**Keywords:** Austrophasmatidae, Asymmetric internal genitalia, Absence asymmetry, False-male-above mating position

## Abstract

**Background:**

Asymmetries are a widespread phenomenon in otherwise bilaterally symmetric organisms, and investigation of asymmetric structures can help us gather insights into fundamental evolutionary processes such as the selection for morphological novelties caused by behavioural changes. In insects, asymmetric genitalia have evolved in almost every order, and usually it’s the sclerotized parts and most conspicuous male phallic organs that are known to exhibit asymmetries. While external copulatory organs in insects have often been subject to investigations concerning asymmetries and the evolution thereof, internal reproductive structures have received far less attention. Here we describe the internal and external male genitalia in three species of Austrophasmatidae, Mantophasmatodea, using μ-CT imaging and light microscopy. Mantophasmatodea is the most recently discovered insect order, and with 21 species described to date, it is among the smallest insect orders currently known.

**Results:**

We confirm that male heelwalkers exhibit asymmetries in the external genitalia and associated structures, represented by asymmetric phallic lobes and cerci. Moreover, we found an extreme asymmetry within the internal male genitalia: in all adult males investigated (*N* = 5), the seminal vesicle, a dilatation of the vas deferens, was only developed on the right side of the male while missing on the left side.

**Conclusion:**

The false-male-above mating position exhibited by Mantophasmatodea and especially the long copulation duration of ca. 3 days might select for this unusual absence asymmetry of the left seminal vesicle. If this holds true for all heelwalker species, this absence asymmetry constitutes another autapomorphy for Austrophasmatidae or even the insect order Mantophasmatodea.

**Supplementary Information:**

The online version contains supplementary material available at 10.1186/s40850-021-00105-6.

## Background

Asymmetries are a common phenomenon in otherwise strictly bilaterally symmetric organisms [1–5]. The investigation of such asymmetric structures can help us gather insights into fundamental evolutionary processes such as the selection for morphological novelties caused by behavioural changes [6]. Animal genitalia represent an important character system to study asymmetries, since they evolved independently multiple times, they are usually quite easily accessible for investigation, and taxonomic studies provide a vast body of data on such structures [6, 7]. In insects, asymmetric genitalia have evolved in almost every order. Usually, the sclerotized parts such as the most conspicuous male phallic organs or aedeagi are known to exhibit asymmetries [6, 7]. Various hypotheses about the evolution of genital asymmetries have been suggested, for example, ecological constraints, internal space constraints that favour asymmetric morphology of internal organs which secondarily affect genitalia, sexual selection via antagonistic coevolution, and sexual selection favouring asymmetry as a mechanical compensation for behavioural changes of the mating position [6, 7]. While external copulatory organs in insects have often been subject to investigations concerning asymmetries and the evolution thereof [8–11], internal reproductive structures have received far less attention. Usually, insect male internal reproductive organs consist of paired (and bilaterally symmetric) mesodermal testes, a pair of lateral ducts, the vasa deferentia, which each contain an enlarged region to serve as a sperm reservoir (vesiculum seminalis), and a median ectodermal ductus ejaculatorius. Ectodermal accessory glands are also commonly present, located at the distal end of the ejaculatory duct ([12]: 573).

**Fig. 1:**
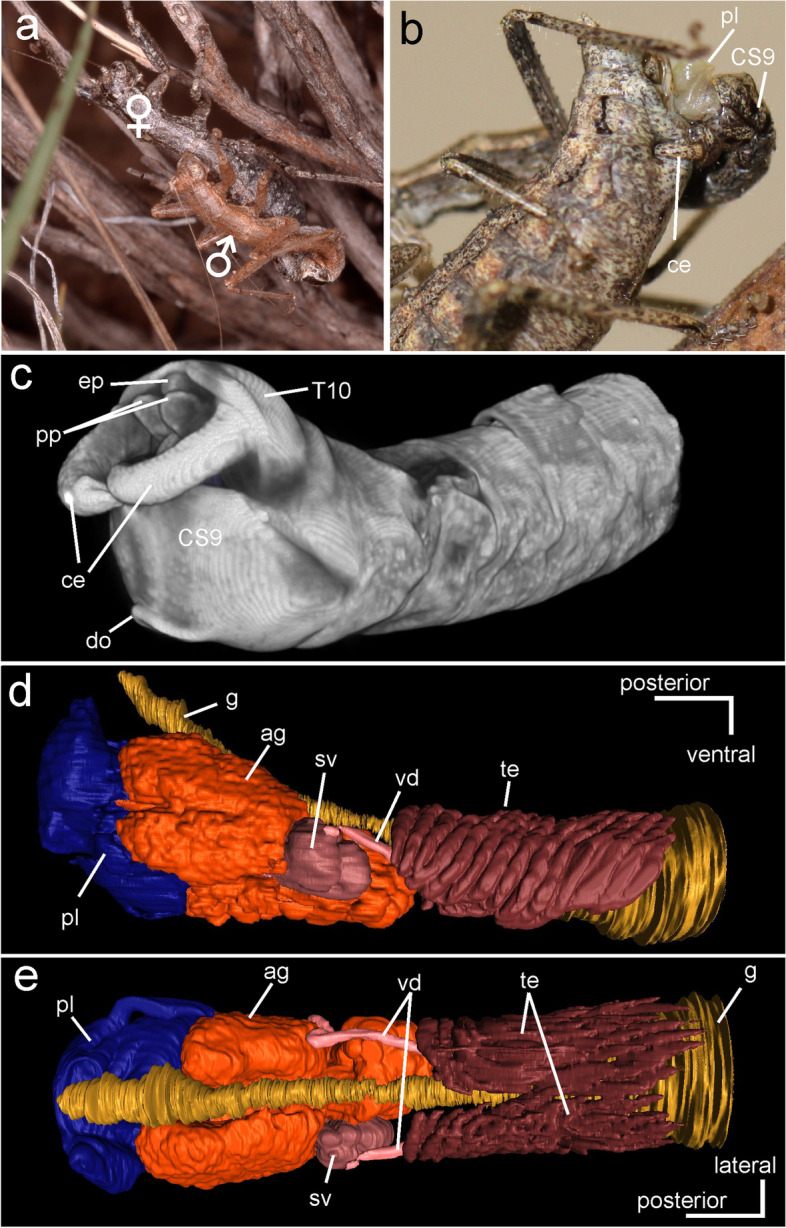
**a** Mating couple exhibiting the false-male-above-mating position in *Karoophasma biedouwense* (Mantophasmatodea). The male sits above the female and bends its abdomen in an S-shape around her right side, bending its abdomen to the left to insert external genitalia into the female genital opening (photo by Mike D. Picker). **b** Close-up of the copulation position during the insertion process. Note the cerci which are used as claspers to hold on to the female abdomen, while the membranous phallic organ is inserted. **c** μ-CT-based volume rendered reconstructed postabdomen of a male *K. biedouwense*. **d-e** μ-CT-based 3D reconstructions of internal reproductive organs and gut from lateral (**d**) and dorsal view (**e**). ag – accessory gland, CS9 – abdominal coxosternum IX (subgenital plate), ce – cerci, do – drumming organ, ep – epiproct, g – gut, pl – phallic lobes, pp – paraproct, sv – seminal vesicle, T10 – abdominal tergum X, te – testis, vd – vas deferens

Mantophasmatodea (heelwalkers, Fig. 1a) are the most recently discovered insect order [13], and with 21 species described to date, they are among the smallest insect orders currently known [14]. Skeletal characters of male and female genitalia have been used for taxonomic studies [15–17]. The male genitalic region of Mantophasmatodea resembles that of many other insect taxa with abdominal segment IX being a highly differentiated genitalic segment [17, 18]. The venter of segment IX functions as a subgenital lobe, covering the external genitalia ventrally. On its outer side, the subgenital lobe of heelwalker males carries a sclerotized median process, used as a drumming organ to produce vibrational signals during courtship [19, 20]. Another special feature of the heelwalker male postabdomen is the so-called vomeroid: a produced transverse sclerite on segment X, which probably serves as an accessory copulatory element but does not directly belong to the genitalia [13, 17]. The external male genitalia of heelwalkers (which are everted during copulation) exhibit strong directional asymmetry since they comprise a large, deeply invaginated left part and a smaller right part ([17]: Fig. 5A therein). Both sides of this phallic tube are predominantly membranous with a complicated structure through the presence of many folds, tendons, apodemes, lobes and sclerites; the latter can actually be used for taxonomical determination of the species [17].

While the outer and inner cuticular ectodermal structures of male genitalia have been extensively described for heelwalkers, hardly any information is available for the internal genitalia and for the tissue underlying the cuticle of the male genitalic region. A general description of the non-cuticular internal parts of the male reproductive organs was outlined in a chromosomal study for *Karoophasma biedouwense* [21]: The reproductive system was described to consist of paired, elongated testes represented by numerous follicles, paired vasa deferentia, a posterior tubular sperm storage organ or seminal vesicle, and tubular paired accessory glands forming an utricular gland.

During copulation, Mantophasmatodea adopt a so-called false-male-above mating position, where the male sits above the female and bends his abdomen sideways around the female so that his genitalia contact the female from below (Fig. 1a,b). Heelwalker males always bend their abdomen around the right side of the female and thus exhibit a one-sided asymmetric mating position [6, 19]. The – in many heelwalker species slightly asymmetric – cerci facilitate the coupling, acting as claspers [19]. Mating and genital coupling in this position can last up to 3 days until the couple separates [14, 22–24].

In the present study, we compare the reproductive organs of males of three species of Mantophasmatodea using X-ray micro-computed tomography (μ-CT) and light microscopy. We present the first detailed description of the non-cuticular and cuticular structures of the male internal reproductive organs, and compare our findings to data on male reproductive organs in other polyneopteran insects, as well as discuss the evolution of asymmetry in internal reproductive organs of heelwalkers.

## Materials and methods

### Specimens

We examined adult males of *Karoophasma biedouwense* Klass et al. 2003, *Karoophasma botterkloofense* Klass et al. 2003, and *Viridiphasma clanwilliamense* Eberhard et al. 2011 (Mantophasmatodea: Austrophasmatidae). The specimens of *K. biedouwense* and *V. clanwilliamense* were collected as nymphs between July and August in 2006 and 2007 at Clanwilliam Dam, Western Cape Province, South Africa (32.213°S, 18.879°E), *K. botterkloofense* was collected in September 2007 near Loeriesfontein, Northern Cape, South Africa (30.974°S, 19.466°E). Juvenile insects were reared to adulthood, subsequently fixed in alcoholic Bouin’s solution for 1–2 days, and stored in 70% ethanol.

### μ-CT and 3D reconstruction

For μ-CT analysis, we used one male *K. biedouwense*. Sample preparation and μ-CT scanning was conducted according to Sombke et al. [25]. The abdomen was cut off, dehydrated in an ascending ethanol series and stained in 1% iodine solution (Carl Roth GmbH + Co. KG, Germany) for 24 h. The sample was then transferred back to 100% ethanol, critical-point dried (Leica EM CPD300, Leica Microsystems, Germany), and mounted on a pin head using glue from a glue gun. The sample was scanned on an Xradia MicroXCT-200 imaging system (Carl Zeiss AG, Germany) with a 4x magnification, 40 kV voltage, 8 W power, and 0.7 s exposure time (180° scan). Resulting pixel sizes were 5.33 μm. Tomography projections were reconstructed using the software XMReconstructor (Carl Zeiss AG, Germany), resulting in an image stack of 995 pictures. Visualization and 3D reconstruction were done with Amira 6.4.0 software (Visualization Sciences Group, FEI Company, OR, USA). Images of the reconstructions were subsequently edited with Adobe Photoshop (CS4, Adobe Systems Inc., USA).

### Histology

For histological sectioning, we used two males of *K. biedouwense* and one male of *V. clanwilliamense* and *K. botterkloofense*, respectively. The abdomens were cut off, dehydrated in an ascending series of ethanol and then embedded into paraffin using three intermediate steps with tetrahydrofuran (Rotipuran®, Carl Roth GmbH + Co. KG, Germany) to facilitate penetration: 2 h 1:1 in ethanol, 24 h pure tetrahydrofuran, and 24 h 1:1 in paraffin [15]. Sections (5 μm thick) were made with a Microm HM 360 rotary microtome (Thermo Fisher Scientific, MA, USA), stained with Azan-novum according to Geidies [26], and analysed using an Olympus BX60 microscope (Olympus, Japan) equipped with a Zeiss AxioCam MRc camera (Carl Zeiss AG, Germany), and AxioVision 4.8 software (Carl Zeiss AG, Germany).

If not stated otherwise, our morphological terminology follows Klass et al. [17] for postabdominal structures and Snodgrass [12] for internal genitalia. All figure plates were arranged using Corel Draw (X7, Corel Corporation). Measurements were conducted on the histological sections using the AxioVision 4.8 software (note that measurements were done on the fixed, embedded and sectioned structures).

## Results

The reproductive organs of male heelwalkers were similar for all three species under investigation (*N* = 5 males) and consisted of paired testes and vasa deferentia, a single seminal vesicle, a median ejaculatory duct, large accessory glands, and a complex phallic organ. To avoid redundancy, all parts are described in detail mainly based on *K. biedouwense* in the following section (histological sections of the other two species are shown in supplementary Fig. S1).

### Testes

**Fig. 2:**
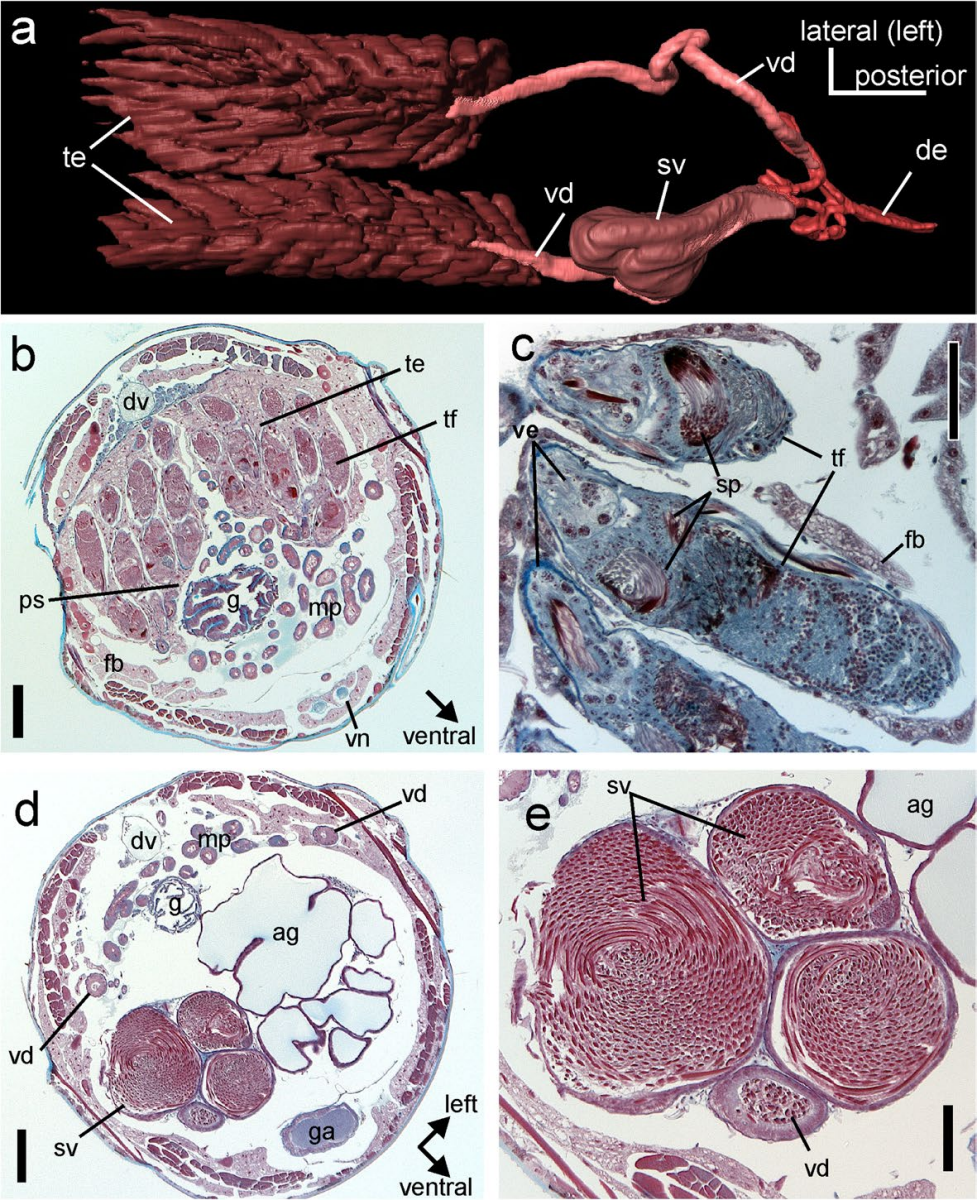
**a** μ-CT-based 3D reconstruction of internal reproductive organs viewed from ventral. Notice that the seminal vesicle (sv) is only developed on the right side. **b** Histological cross section of *K. biedouwense* male postabdomen, approximately through abdominal segment VI; bar = 200 μm. **c** Close-up of testicular follicles from horizontal section through *K. biedouwense* male postabdomen, bar = 20 μm. **d** Cross section through *K. biedouwense* at the location of the seminal vesicle, bar = 200 μm. **e** Close-up of seminal vesicle, bar = 100 μm. ag – accessory gland, de – ductus ejaculatorius, dv – dorsal vessel, fb – fat body, g – gut, ga – ganglion, mp – Malpighi vesicles, ps – peritoneal sheath, sv – seminal vesicle, sp – spermatozoa, te – testis, tf – testicular follicle, vd – vas deferens, ve – vas efferens, vn – ventral nerve cord

In the male specimen of *K. biedouwense* that we scanned by μ-CT, the testes were located in abdominal segments IV – VI, and situated laterodorsally of the gut (Fig. 1d, e). Each testis consists of approximately 30 testicular follicles that open independently into the vas deferens via a very short vas efferens. The wall of the follicles is a thin, one-layered epithelium (2–6 μm thick) and all follicles of a testis are surrounded by a peritoneal sheath embedded in fat body (Fig. 2b). The testes meet dorsally to the gut at the midline, but there’s no detectable fusion or common peritoneal sheath (Fig. 2b). Within each testicular follicle, sperm of various developmental levels are found, with the most mature spermatozoa being located at the posterior ends of the follicles in proximity to their exit ducts (Fig. 2b,c).

### Vasa deferentia and seminal vesicle

The vasa deferentia lead posteriorly from the testes, the tubular ducts consist of a quite thick cellular epithelium (19–25 μm thick; Fig. 2a, d, e). Within the VII - VIII^th^ abdominal segments, the vasa deferentia on both sides bend dorsally and then coil back ventrally (Fig. 2a). Only on the right side of the male, the vas deferens is enlarged, constituting a wound-up tubular seminal vesicle filled with mature sperm (Fig. 2a, d, e). The seminal vesicle exhibits a thinner epithelial wall than the tubular vas deferens (6–9 μm thick; Fig. 2e). In all five male specimens investigated here, only the seminal vesicle on the males’ right hand side was developed, while the vas deferens on the left hand side did not exhibit any enlargements or changes in the epithelial wall or muscle fibre surrounding (Fig. 2a, d, e). Both vasa deferentia are connected to the ejaculatory duct, approximately within the IX^th^ abdominal segment. No cuticular lining was found within the lumen of the vasa deferentia.

### Ductus ejaculatorius

**Fig. 3:**
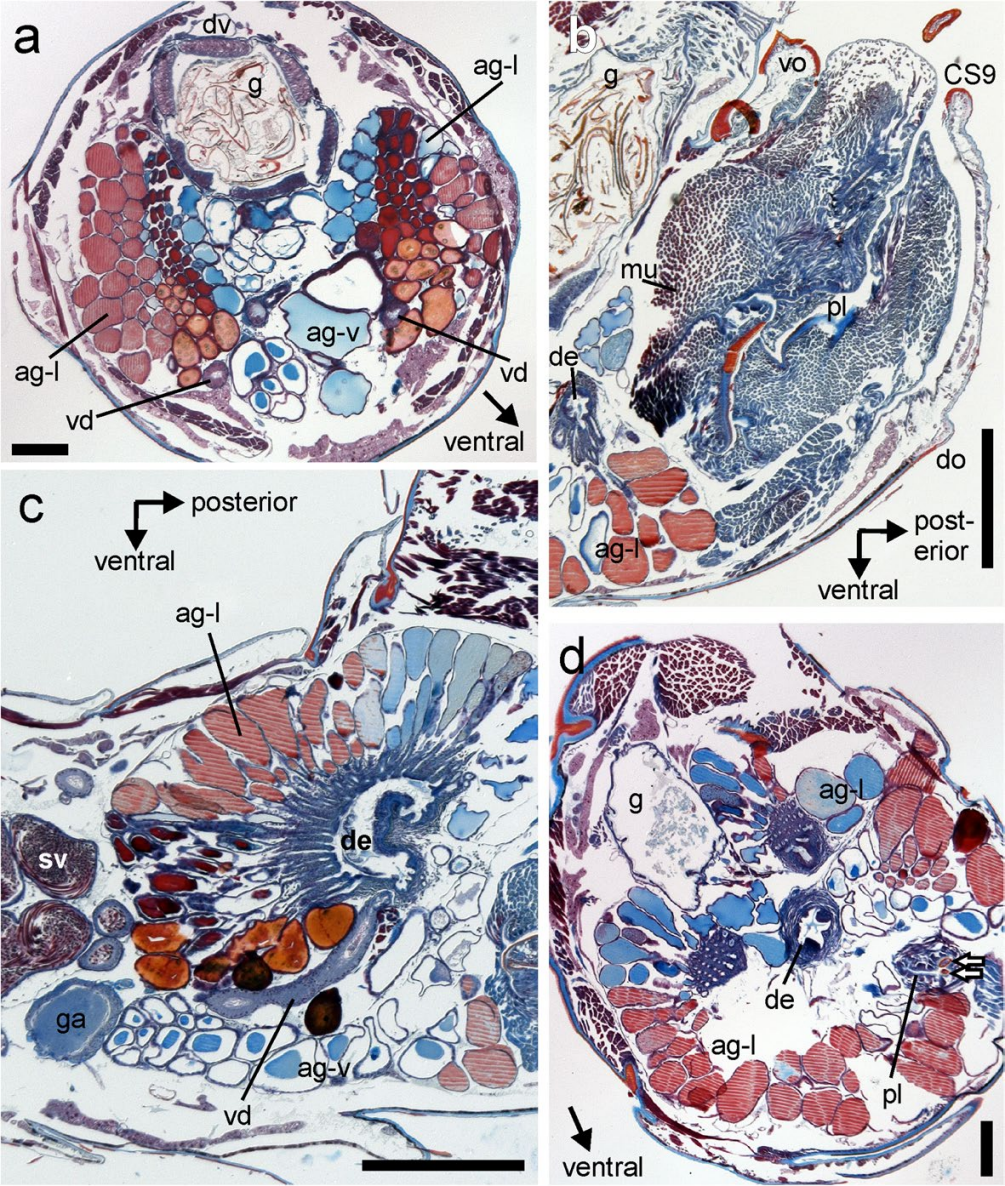
**a** Histological cross section of *K. biedouwense* male postabdomen, through the VIIIth segment; bar = 200 μm. **b** Sagittal section through abdominal tip of *K. biedouwense* postabdomen; bar = 500 μm. **c** Sagittal section through abdominal segments VII-IX showing insertion of tubular accessory glands into ductus ejaculatorius; bar = 100 μm. **d** Cross section through IXth abdominal segment, with the most internal portion of the phallic tube visible – sections of the slcerite hooks phl and phs visible (arrows); bar = 200 μm. ag-l – lateral accessory gland, ag-v – ventral accessory gland, CS9 – subgenital plate, de – ductus ejaculatorius, do – drumming organ, dv – dorsal vessel, g – gut, ga – ganglion, mu – musculature, pl – phallic lobes, sv – seminal vesicle, vd – vas deferens, vo – vomeroid

The unpaired, median ductus ejaculatorius receives both vasa deferentia as well as the openings of the accessory glands within the IX^th^ abdominal segment and leads posteriorly to the outside, ventrally to the gut. At the insertion site of the accessory glands, the ejaculatory duct is forked (Fig. 2a). The inner wall of the posterior-most section of the ductus ejaculatorius is lined with cuticle and its epithelial wall is surrounded by a strong muscular sheath, mainly consisting of circular muscle fibres (Fig. 3d).

### Accessory glands

The accessory glands arise from the anterior end of the ejaculatory duct, which forms short divergent anterior branches (Fig. 3c). The accessory glands appear in three large compartments, each consisting of numerous elongated and wound-up tubular glands, consisting of a thin single layer of epithelial cells (4–6 μm thick). In the μ-CT-reconstruction of *K. biedouwense*, we found one accessory gland-compartment located ventrally to the vasa deferentia and gut, at the ventral midline of the abdominal segments VI to VIII (Fig. 1d, e). This compartment had large vesicular tubes with clear lumina or filled with bluish secretion in the histological sections (Figs. 2d, 3a, c). Two morphologically similar accessory gland compartments are located to the left and right sides of the ejaculatory duct within abdominal segments VII-X. Both compartments consist of numerous tubules filled with differently staining secretions (Fig. 3). All glandular tubules open into the ejaculatory duct (Fig. 3c), no cuticle was found to line the lumen of the ducts.

### Phallic lobes

The membranous cuticular details of the phallic lobes of heelwalker males have been extensively described by Klass et al. [17], so only a short repetition is given here: Both sides of the phallic tubes are predominantly membranous with a complicated structure through the presence of many folds, tendons, apodemes, lobes and sclerites. They exhibit strong directional asymmetry since they comprise a large, deeply invaginated left part and a smaller right part ([17]: Fig. 5A therein). Due to the membranous, wound-up structure of the phallic lobes and the low resolution of our μ-CT scan it was impossible to reconstruct the phallic lobes’ membranes in detail. Both in the histological sections and μ-Ct-scan muscle tissue was found associated with the cuticular membranes, filling up most of the volume inside the abdominal tip underneath the subgenital plate (Fig. 3b). The gonopore, i.e. the opening of the ejaculatory duct to the outside lies within the membranous folds of the phallic lobes, which subsequently open into the space above the subgenital plate (Fig. 3b).

## Discussion

In this study we show that the male internal genitalia of Mantophasmatodea basically follow the common organization found in other polyneopteran insects ([12]: 573ff) by consisting of paired, follicular testes, paired vasa deferentia which open ventrally into the unpaired ductus ejaculatorius, where accessory glands are attached. However, the organization of the seminal vesicles, which are only developed at the right hand side of each adult male investigated so far, represents a feature unique to Austrophasmatidae (Mantophasmatodea).

Follicular testes are typically found in insects and the number of testicular follicles can range from only one to more than 100 [27]. Numerous follicles were reported for Phasmatodea, Blattodea, Mantodea and Orthoptera [27]; the approximately 30 follicles per testis found in the three Austrophasmatid species investigated here also fall in this range. The various developmental stages of spermatozoa found within the testicular follicles (Fig. 2b, c) indicates that both testes are functional. A similar organization of series of testicular follicles that open individually into the vas deferens via a short vas efferens is e.g. known from Orthoptera [27]. The paired vasa deferentia did not exhibit any obvious differences from the insect groundplan. In contrast, the seminal vesicle, which is usually found on both sides as a dilatation of the vasa deferentia, exhibited a unique asymmetry with only the right vesicle developed. All adult heelwalker males investigated here (*N* = 5) did not have a seminal vesicle on the left side, while the testis and vas deferens were intact and functioning. Moreover, the vas deferens on the left side did not show any indication of a temporal dilatation (such as e.g. a thinner epithelium). We therefore conclude that male heelwalkers of the family Austrophasmatidae do not possess a seminal vesicle on their left side. In insects, the seminal vesicles are used to store fully developed sperm before transfer to the female. In some insects such as Orthoptera, some Odonata and Coleoptera, seminal vesicles are not dilatations of the vas deferens but separate structures. In Austrophasmatidae, the vasa deferentia and the single seminal vesicle are not lined with cuticle (i.e. they are of mesodermal origin). The seminal vesicle on the right hand side of the male was a large dilatation of the vas deferens and packed with mature sperm (see Fig. 2e). The fine structure of the male sperm was described by Dallai et al. [28], using *Mantophasma zephyrus,*^1^ however, they did not mention the unusual organisation of the seminal vesicles therein. Presumably, all mature sperm from both testes is stored in the single seminal vesicle, so all mature sperm from the left testis travels to the right vesicle via the vas deferens. How this is achieved is not known so far. Alternatively, mature sperm from the left testis might be transported directly to the ejaculatory duct during copulation without temporary storage. However, mature spermatozoa are reported to form spermatodesms (bundles of connected spermatozoa) within the seminal vesicles [28] and are then transferred to the female as a package during copulation to ensure a transfer of a high number of sperm. So a direct transfer of single spermatozoa from the left testis to the female seems unlikely. From the anatomy of the testes and other structures of the male internal genitalia involved in sperm production, transfer, and storage, this question cannot be answered currently.

The accessory glands in Mantophasmatodea males arise from tubular diverticles of the anterior mesodermal part of the ejaculatory duct, constituting so-called mesadenia, a structure common to orthopterans and orthoperoid insects [27]. Especially the lateral accessory glands exhibit at least four different areas with differently staining secretion in the histological sections, which hints towards different pH levels (Fig. 3). Such a regional differentiation within glandular compartments is also known from e.g. Coleoptera and Lepidoptera [27]. Secretions of the accessory glands are most probably involved in producing a spermatophore during copulation, providing nourishment and protection to the sperm [27]. An observational study on an austrophasmatid species showed that copulating males did not lose more weight during mating than control males that were not fed for the same amount of time [23]. The sperm and glandular secretion transferred to the female during the elongated copulation in Mantophasmatodea, which can last up to 3 days, therefore does not seem to involve a great amount of fluids, at least in terms of mass of body fluid transferred. Secretions from the accessory glands could also change the behaviour of the female after copulation, e.g. to inhibit re-mating with other males [29]. Female heelwalkers did not respond to vibratory male signals after they had mated (Eberhard, personal observation), but if male seminal fluids are responsible for this behavioural change has not been tested yet.

The asymmetry of the phallic lobes of male Mantophasmatodea, with the left part being much larger and complex than the right part has been described by Klass et al. [17] and reported in Huber et al. [6]. Asymmetries in external genital morphology are known in several animal taxa and often seem to be related to lateralised mating behaviour [5, 30]. However, the relationship between genital asymmetry and lateralised mating behaviours, as well as their evolutionary significance, has rarely been examined. In insects, genital asymmetries are very common and widespread, and have probably arisen independently from symmetrical structures in several taxa. The mating position exhibited by the respective insect taxa was hypothesised to be the main driving force for the evolution of genital asymmetry in insects [7, 31]. For example, some apparently symmetric Heteroptera and Dermaptera have a preference for mating via one side [6, 32]. This suggests that the behavioural asymmetry pre-dates the morphological asymmetry. An important potential consequence of twisting or asymmetrical flexing of the abdomen due to a change in mating position is that male and female genitalia do not contact symmetrically any more. The compensation for such asymmetric contact may be one of the major selective forces driving the evolution of genital asymmetry [6]. While the lateralised mating position found in Mantophasmatdoea might have led to the evolution of the asymmetry in phallic lobes and male cerci, the extreme asymmetry found in the internal genital organisation needs a different explanation.

Asymmetry resulting from complete reduction of one side of external reproductive structures are known in cimicoid bugs, Eudermaptera, and possibly Zoraptera, where one of the two penis lobes are completely reduced [6]. In some Theridiidae spiders, absence asymmetry results from amputation of one palp (the sperm transferring structures) after the penultimate moult [6, 33]. Such an asymmetric loss may not be advantageous per se, or it may be even disadvantageous in respect to sperm transfer, but material, energy, and development time may be saved [6]. Asymmetry in internal bilaterally organized organs has rarely been investigated. Complete absence of one organ of a bilateral pair, also called absence asymmetry, seems to be quite rare among animals. Among vertebrates, such losses are largely restricted to snakes or snake-like animals, where one of bilaterally paired lungs has been reduced [34], and birds, where loss of one ovary is common [35]. In insects, absence asymmetry is found only in some Coleoptera and Hemiptera, where ovaries are symmetrically lost in aphids and some scarab beetles, and asymmetrical monorchy, i.e. the complete absence of one testis, was reported in 174 species of carabid beetles [36]. In these species, reduction of one of the bilaterally symmetrical internal organs was hypothesised to concur with small body sizes and hence visceral packing constraints [36]. In the comparatively large Mantophasmatodea, the absence of one seminal vesicle coupled with the presence of its bilateral counterpart while both testes are similarly well developed, seems to be a special feature. All adult males investigated so far (*N* = 5) had well-developed paired testes, but only one seminal vesicle on the right side, while no seminal vesicle could be found on the left side. The loss of the left seminal vesicle could have arisen as a mechanical compensation to the mating position in heelwalkers. During copulation, males need to bend their abdomen around the right side of the female (Fig. 1a) and thus exhibit a one-sided asymmetric mating position [6, 19]. Of course, this extremely flexed position of the abdomen maintained during the lengthy mating duration could cause the left side of the male with all internal organs to be squished together due to the intense folding of the body, while the right side is not as affected. Due to the mating position, a seminal vesicle on the left side of the male might not be functional any more during copulation.

## Conclusions

We described the internal genital organisation of male Mantophasmatodea in three austrophasmatid species and revealed an absence asymmetry of the seminal vesicles in the species under investigation. If this holds true for all heelwalker species, this character constitutes another autapomorphy for Austrophasmatidae or even the insect order Mantophasmatodea. The false-male-above mating position and elongated copulation duration might be responsible for the asymmetries found in male external and internal genital structures. Presumably, asymmetry in external genital structures evolved due to the asymmetric coupling of male and female genitalia [7], and the absence asymmetry of the seminal vesicles in heelwalkers probably evolved due to space constraints within the twisted male abdomen during copulation.

## Supplementary Information

**Additional file 1: Supplementary Figure S1.** Histological sections of *Viridiphasma clanwilliamense* (a-d) and *Karoophasma botterkloofense* (e,f) male postabdomen*.* a) Cross section through Vth abdominal segment at the location of the testes. b,c) Cross sections at the location of the seminal vesicle; section in (c) slightly tilted horizontally. d) Horizontal section through abdominal segments VIII-IX showing insertion of tubular accessory glands into ductus ejaculatorius. e) Horizontal section through VII – VIIIth abdominal segments showing sections of testes, vas deferens and seminal vesicle. f) Horizontal section through abdominal tip showing sections of phallic tube and associated musculature. Note that fixation and/or staining for *K. botterkloofense* specimen was not ideal, so the sections appear greyish and torn at some locations; however, the main compartments of the postabdomen could be followed and reconstructed without doubt. ag-l – lateral accessory gland, ag-v – ventral accessory gland, CS9 – subgenital plate, de – ductus ejaculatorius, dv – dorsal vessel, g – gut, ga – ganglion, mp – Malpighi vesicles, mu – musculature, pl – phallic lobes, sv – seminal vesicle, te – testis, tf – testicular follicle, vd – vas deferens, vn – ventral nerve cord.

## Data Availability

Datasets used during the current study are available from the corresponding author upon reasonable request.

## Footnotes

1 Originally, the species was named *M. zephyra*, but Klass et al. (2003) described the Etymology as “Latin for west wind”, so we consider M. *zephyrus* to be the correct species name according to ICZN Art. 11.9 and Art.31.2.1
