# Supplementary figures and images for: Asymmetry of the male internal reproductive organs in Mantophasmatodea
Source: BMC Zool. 2022 Jan 7;7:4. doi: 10.1186/s40850-021-00105-6 (PMC10127319; doi:10.1186/s40850-021-00105-6)

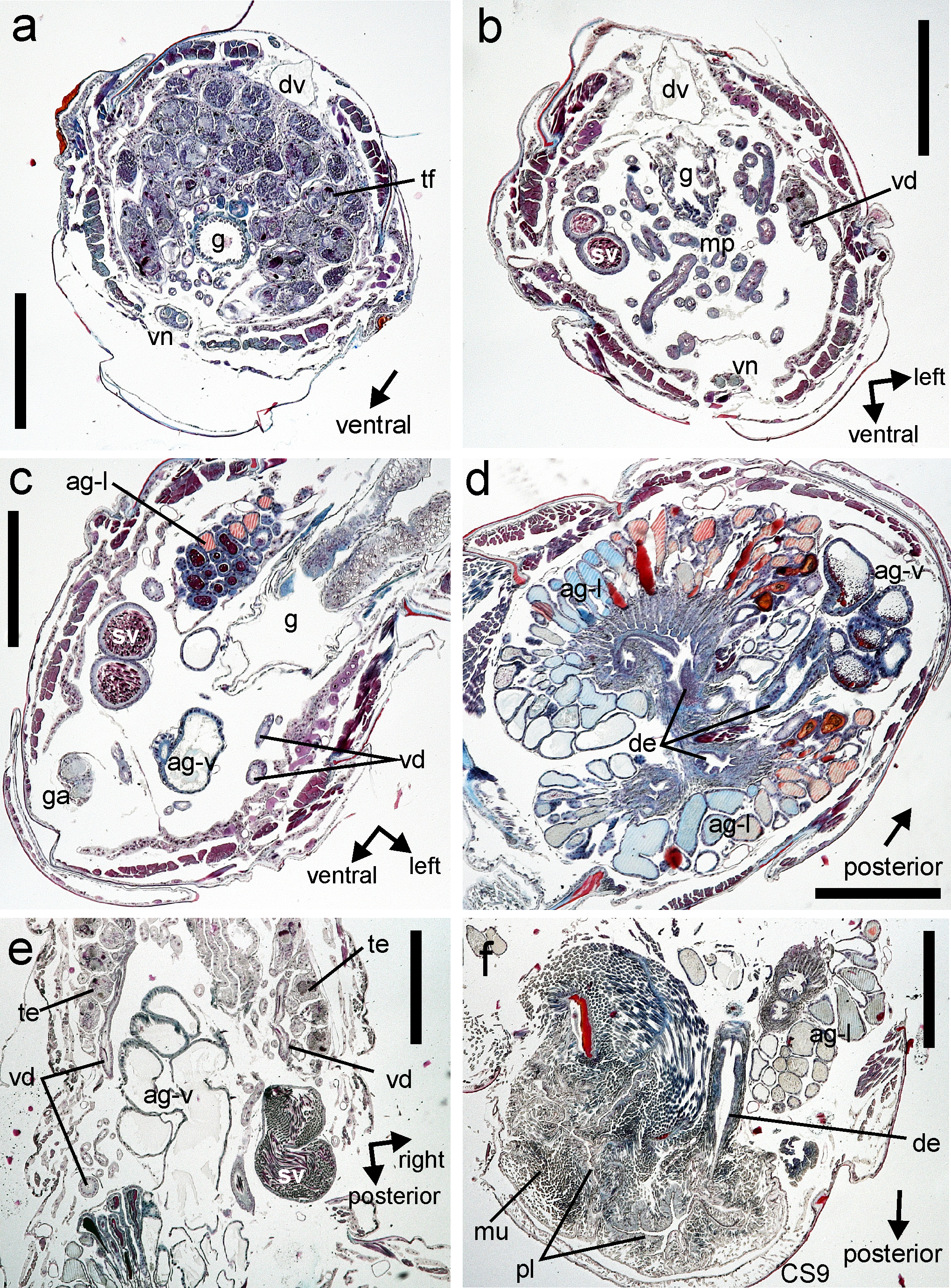

Supplement: Supplementary file 1 — Additional file 1: Supplementary Figure S1. Histological sections of Viridiphasma clanwilliamense (a-d) and Karoophasma botterkloofense (e,f) male postabdomen. a) Cross section through Vth abdominal segment at the location of the testes. b,c) Cross sections at the location of the seminal vesicle; section in (c) slightly tilted horizontally. d) Horizontal section through abdominal segments VIII-IX showing insertion of tubular accessory glands into ductus ejaculatorius. e) Horizontal section through VII – VIIIth abdominal segments showing sections of testes, vas deferens and seminal vesicle. f) Horizontal section through abdominal tip showing sections of phallic tube and associated musculature. Note that fixation and/or staining for K. botterkloofense specimen was not ideal, so the sections appear greyish and torn at some locations; however, the main compartments of the postabdomen could be followed and reconstructed without doubt. ag-l – lateral accessory gland, ag-v – ventral accessory gland, CS9 – subgenital plate, de – ductus ejaculatorius, dv – dorsal vessel, g – gut, ga – ganglion, mp – Malpighi vesicles, mu – musculature, pl – phallic lobes, sv – seminal vesicle, te – testis, tf – testicular follicle, vd – vas deferens, vn – ventral nerve cord. [file 40850_2021_105_MOESM1_ESM.tif]
